# Effects of the CO_2_ Guest Molecule on the *sI* Clathrate Hydrate Structure

**DOI:** 10.3390/ma9090777

**Published:** 2016-09-15

**Authors:** Fernando Izquierdo-Ruiz, Alberto Otero-de-la-Roza, Julia Contreras-García, Olga Prieto-Ballesteros, Jose Manuel Recio

**Affiliations:** 1 Departamento de Química Física y Analítica, Universidad de Oviedo, Oviedo 33006, Spain; jmrecio@uniovi.es; 2 Centro de Astrobiología (INTA-CSIC), Torrejón de Ardoz 28850, Spain; prietobo@cab.inta-csic.es; 3 Department of Chemistry, University of British Columbia, Kelowna, BC V1V1V7, Canada; aoterodelaroza@gmail.com; 4 Laboratoire de Chimie Théorique, CNRS & Université Pierre et Marie Curie, Sorbonne Universités, Paris 75005, France; Julia.Contreras@lct.jussieu.fr

**Keywords:** clathrates hydrates, carbon dioxide, DFT, high pressure

## Abstract

This paper analyzes the structural, energetic and mechanical properties of carbon dioxide hydrate clathrates calculated using finite cluster and periodic ab initio density-functional theory methodologies. Intermolecular interactions are described by the exchange-hole dipole moment method. The stability, gas saturation energetics, guest–host interactions, cage deformations, vibrational frequencies, and equation of state parameters for the low-pressure *sI* cubic phase of the CO_2_@H_2_O clathrate hydrate are presented. Our results reveal that: (i) the gas saturation process energetically favors complete filling; (ii) carbon dioxide molecules prefer to occupy the larger of the two cages in the *sI* structure; (iii) blue shifts occur in both the symmetric and antisymmetric stretching frequencies of CO_2_ upon encapsulation; and (iv) free rotation of guest molecules is restricted to a plane parallel to the hexagonal faces of the large cages. In addition, we calculate the librational frequency of the hindered rotation of the guest molecule in the plane perpendicular to the hexagonal faces. Our calculated spectroscopic data can be used as signatures for the detection of clathrate hydrates in planetary environments.

## 1. Introduction

Clathrate hydrates are of primary importance in a variety of fields, from life sciences to planetology, and constitute a natural resource in the energy industry. In environmental science, the formation of clathrates has been proposed as a method to effectively store greenhouse gases by injecting volatiles into potential deep hydrate-forming deposits [1,2]. These crystalline compounds accommodate guest molecules (usually a non-polar gas) within the framework of a host three-dimensional network of water cages or channels. Although it ultimately depends on the nature of the guest molecule, clathrate hydrates generally need high pressure and low temperature to be stable. Pressure and temperature stability ranges are wide, displaying pressure-induced phase transitions between the main types of known clathrate hydrate structures: cubic *sI* and *sII*, hexagonal *sH*, and orthorhombic FIS (Filled Ice Structure). The study of the high pressure (HP) phases of these clathrates is particularly relevant in planetary geophysics applications. Giant moons, such as Ganymede or Titan, show evidence of having deep internal water-rich layers in several phases, including liquid water layers [3]. Some structural models indicate that liquid layers constitute planetary oceans squeezed between different phases of water ice, which might be under pressures up to 1 GPa. It is proposed that methane HP clathrates can be present in Titan below its ocean [4]. If CO_2_ is present within icy bodies like Ganymede or Pluto, it should form HP clathrate hydrates as well [5,6]. Their physicochemical properties, different from those of water ice phases, affect the thermal state, structure, and dynamics of these planetary objects.

Knowledge of basic structural and energetic behavior has been well established for CO_2_ clathrate hydrates (see for example [7,8] and references therein). However, the low pressure *sI* phase of the CO_2_ hydrate has not received enough attention in the past in contrast with the number of experimental and theoretical studies on methane hydrate clathrates [9]. In recent years, new studies have tried to characterize and interpret its high pressure behavior [6,10] and the kinetics of its formation [11,12]. The ability of the two water cage types in the cubic *sI* phase to host a CO_2_ molecule has been thoroughly examined by Srivastava and Sastry [13] using cluster models. Studies of the energetics involved in the guest saturation process of CO_2_ clathrates provide a wealth of valuable information due to their relevance for storage and environmental purposes, and we believe this topic still deserves a deeper understanding. CO_2_ vibrational and rotational frequencies, structural distortions and guest–host interactions are all affected by the size and shape of the two cages in the *sI* phase of the CO_2_ clathrate hydrate. The comparison between methane and carbon dioxide clathrates is worth exploring as well.

It is our aim to provide a rigorous description of structure and bonding in the *sI* phase of CO_2_@H_2_O. The search for characteristic properties (e.g., vibrational frequencies) of CO_2_@H_2_O clathrates that enable their experimental identification by spectroscopic means is considered. In particular, we predict spectroscopic quantities related to the atomic displacements of the CO_2_ guest molecule inside the clathrate cages. Mass density is also a relevant property of these systems, which we evaluate in the limits of empty and fully occupied states. In addition, the application of pressure is used to analyze the interplay between structure and intermolecular interactions in these systems. Pressure will also allow us to discuss the structural anisotropy of the *sI* phase, to evaluate its influence on the process of gas saturation and to calculate the evolution of guest vibrational frequencies under hydrostatic compression. Differences between carbon dioxide- and methane-containing hydrate clathrates will be discussed as well. New techniques are applied to the analysis of intermolecular interactions in this clathrate, such as the microscopic partition of its equation of state, the topological analysis of non covalent interactions and the proposal of indexes to measure pressure- and guest-induced cage distortions and anisotropy in the CO_2_@H_2_O *sI* structure. After the computational details are introduced in the next section, our results are presented and discussed following seven subsections that cover structure, equation of state, cage distortions, guest molecule orientations, cage occupation, guest–host interactions, and vibrations. The paper ends with a summary of the main conclusions of our study.

## 2. Materials and Methods

Our reference geometry for the host cubic *sI* structure of CO_2_@H_2_O (space group $Pm\bar{3}n$) is taken from the review of Loveday and Nelmes [9]. Due to proton disorder, only oxygen positions were taken into account for the initial skeleton. Hydrogen atoms were added afterwards following the ice rules [14] and trying to minimize the loss of symmetry. Our starting point is one out of the more than 50,000 configurations that yield a negligible dipole moment [15] of the unit cell. In a recent study, Raza et al. [16] showed that the differences in the lattice energies of all possible single-cell proton configurations in cubic and hexagonal ice are in the range of kJ/mol per water molecule. Although the case of the CO_2_-water system is not considered in these works, we expect that a change in proton configuration will not have a significant impact on guest–host interactions. We also notice that Takeuchi et al. [15] have recently explored the energetic landscape of proton configurations in clathrate hydrates. Differences in total energies were found to be in the order of 0.3 kJ/mol and the “energies of the guests in the different cages vary by 0.021 kJ·mol^−1^ and 0.465 kJ·mol^−1^ for the small and large cages, respectively” ([15], p. 6). Using the lowest energy configuration from the work of Takeuchi et al., we have double checked some of the calculations to verify that our results are not affected by the choice of proton ordering.

**Figure 1:**
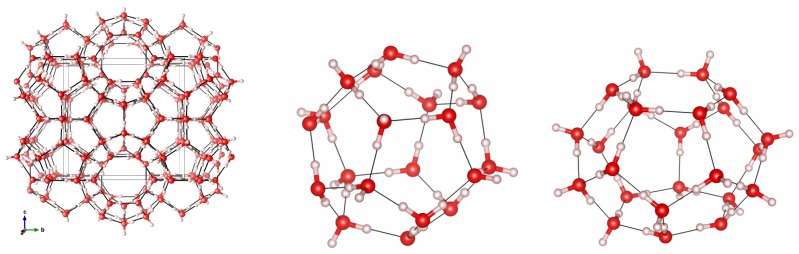
Unit cell (**left**) and P (**center**) and H (**right**) types of cages in the *sI* structure. Oxygen is shown in **red** and hydrogen is in **white**.

The *sI* structure has 46 water molecules that forms eight different cages per unit cell. Two cages are smaller than the other six. The former will be denoted as 5^12^ or “P” cages because they are formed by 20 water molecules arranged in a stretched dodecahedron with twelve pentagonal faces. The other six cages are larger in volume, and will be denoted as 6^2^5^12^ or “H” cages because they have two hexagons in their structure. In Figure 1, both types of cages and the unit cell for the *sI* structure are shown. Guest carbon dioxide molecules are arranged in their usual linear configuration with the carbon atoms positioned approximately at the center of the cages. These guest molecules are not displayed in Figure 1. Their preferred orientation will be discussed in further detail in the remainder of this article.

Density functional theory (DFT) calculations were performed in both finite cluster models and in the periodic cubic *sI* structure at different unit cell geometries. In the latter case, the plane-wave/pseudopotential approach within the framework of the Projector Augmented Wave (PAW) method [17], as implemented in the Quantum Espresso code [18] (version 4.3.2), was used with the following computational parameters: a cutoff energy of 60 Ry for the plane-wave expansion of the wavefunctions, a Monkhorst–Pack [19] grid of 2 × 2 × 2 *k*-points and the PW86PBE [20,21] Generalized Gradient Approximation (GGA) exchange-correlation functional, which gives an accurate description of the Pauli repulsive wall of non covalent interactions (NCIs) [22,23]. To evaluate NCIs accurately, we used the XDM (exchange-hole dipole moment) dispersion energy model [24,25], which has previously proven to be reliable in equilibrium and non-equilibrium geometries [26]. The XDM correction uses two damping function parameters that we chose as $a_{1}$ = 0.136 and $a_{2}$ = 3.178 Å according to Reference [25]. The accuracy of DFT-XDM for calculations of intermolecular interactions has been established in the past [27,28,29,30]. Binding energies of gas-phase dimers are calculated with an accuracy of 10% or less. Lattice energies of molecular crystals can be calculated to slightly below 1 kcal/mol per molecule.

The cubic unit cell lattice parameter and the atomic positions were optimized first at zero pressure in both the empty host lattice (the water network only; no guest molecules) and in the fully occupied clathrate structure with one CO_2_ molecule in each of the eight cages. Pressure effects were studied by performing a series of periodic calculations in which we fixed the volume of the cell and relaxed the atomic positions. We took several volumes around the equilibrium value at zero pressure. In these calculations, pressure (*p*) was obtained from the calculated stress tensor at the corresponding volume (*V*). The final set of *V*-*p* data points is the input for an equation of state (EOS) fitting using the Vinet equation [31]:

(1) $$p=3B_{0}\left(\frac{1-x}{x^{2}}\right)e^{\frac{3}{2}\left(B_{0}^{\prime }-1\right)\left(1-x\right)};\;x={\left(\frac{V}{V_{0}}\right)}^{\frac{1}{3}},$$

where $B_{0}$ and $B_{0}^{\prime }$ are the zero pressure bulk modulus (inverse of the compressibility) and its first pressure derivative, and $V_{0}$ is the volume at zero pressure. These EOS parameters correspond to static conditions—zero temperature and no vibrational effects.

Following crystal chemistry structure-property analysis, we decomposed the compressibility of the clathrate hydrates into local polyhedral contributions [32]. In CO_2_@H_2_O clathrates, the unit cell is partitioned into discrete non-overlapping water cages. We would like to stress that cage volumes are difficult to evaluate in a manner such that their sum reproduces the total unit cell volume. Conventional computational packages often provide polyhedra volumes, but these do not meet this requirement due to the lack of convexity of the cages. To address this problem, we used an algorithm that averages the minimum and maximum possible volumes for each particular cage. The mean volumes for the two P and six H cages at the calculated pressures were obtained and fitted with the Vinet equation of state. In this way, EOS parameters for each type of cage were calculated.

Next, we carried out a study on how the orientation of the CO_2_ guest molecule affects the energetics of the two types of cages. For this, we built a three-dimensional grid of angle values to sample the possible orientations of a CO_2_ molecule around the position of the carbon atom located at the center of the cage. In these periodic calculations of the *sI* CO_2_@H_2_O phase, we relax the atomic coordinates but not the unit cell. This allows us to find the most favorable orientation inside each of the cages and to obtain a libration frequency related to the hindered rotation of the CO_2_ molecule inside the H cages that will be discussed below. For the evaluation of the libration frequency, we used a torsional harmonic oscillator model:

(2) $$E\left(\phi ;\theta \right)=\frac{1}{2}k\left(\theta \right)\phi^{2};\;k\left(\theta \right)={\left(\frac{\partial^{2}E\left(\phi ;\theta \right)}{\partial \phi^{2}}\right)}_{\theta };\;\nu =\frac{1}{2\pi c}\sqrt{\frac{k}{I}}.$$

In the equation above, *ϕ* stands for the libration angle and *θ* is a parameter related to the orientation of the molecule in the free rotation plane, parallel to the hexagonal face (see Results section). *k* is the force constant and *I* is the moment of inertia of the CO_2_ molecule. The libration is obtained by averaging the different values obtained for several values of *θ*.

After determining the optimal molecular orientation inside each cage, calculations were performed in which we increased the number of cages occupied by a single molecule, from one cage to eight (saturation), and then relaxing the system in each case, with the aim of obtaining the stable cage occupation number.

As clathrate hydrates are bound by non-covalent interactions, we also evaluated the reduced electron density gradient (RDG) [33] in order to highlight the non-covalent interactions within the cages. The RDG is defined as follows:

(3) $$RDG=s=\frac{1}{2{\left(3\pi^{2}\right)}^{1/3}}\frac{\left|\nabla \rho \right|}{\rho^{4/3}},$$

with *ρ* being the electron density at a given point of the space. We used the NCIPLOT keyword in the CRITIC2 program (version 1.0) [34,35], a computational tool that performs topological analyses of different electron-density-related functions in solids. Isosurfaces of the *s* function, colored using the sign of the second eigenvalue of the electron density Hessian times the value of the electron density at each point of the space (sign$(\lambda_{2})\rho$), are useful in order to find the type and strength of the interactions. The coloring scheme is as follows: blue is used for attractive interactions that are related to $\lambda_{2}<0$ and high values of *ρ* typical of hydrogen bond interactions, red is used for repulsive interactions where *ρ* is also relatively large, but $\lambda_{2}$ is positive as in steric repulsions, and finally, green for dispersion interactions with small values of the electron density.

**Figure 2:**
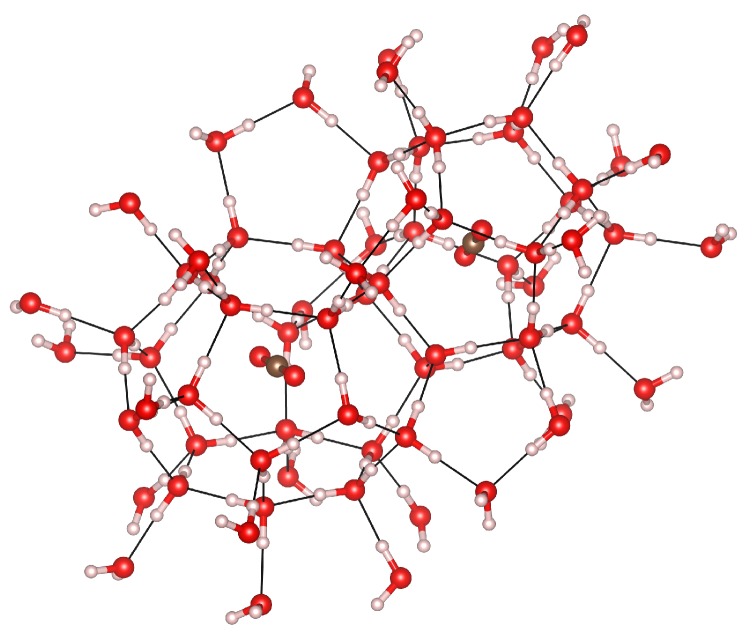
Geometry of the cluster model used in the vibrational calculations taken from the optimized crystalline structure.

Due to the large number of atoms in the unit cell, phonon calculations are very time-consuming for these systems. Cluster calculations constitute a reasonable and accurate alternative due to the local nature of the guest vibrational modes, which are the most representative in the search for characteristic spectroscopic signatures of this gas clathrate hydrate system. We used the Gaussian09 code [36] to obtain vibrational frequencies using the geometries obtained from the periodic calculations. The popular B3LYP hybrid functional [37,38] was used due to its overall good performance, combined with the aug-cc-pVDZ basis set [39]. Vibrations were computed in the harmonic approximation, using a cluster formed by one P and one H cage sharing a pentagonal face (see Figure 2) mimicking the atomic arrangement in the crystal. In all these cluster calculations, the geometries were taken from the optimized crystalline structures obtained at different pressures.

All molecular and crystal representations were performed using the VESTA program (version 3.3.2) [40].

## 3. Results and Discussion

### 3.1. Geometry Optimization and Structural Properties at Zero Pressure

The geometry relaxation of the cubic structure give a unit cell lattice parameter of *a* = 11.642 Å for the equilibrium geometry of the saturated CO_2_@H_2_O *sI* phase, and *a* = 11.673 Å for the empty clathrate. These values are in good agreement with the experimental lattice parameter (11.83 Å) obtained by Henning at 77 K [7]. The experimental lattice parameter is slightly higher than our calculated value, which can be ascribed to thermal expansion. Using the data collected by Udachin et al. [8], we extrapolate the lattice parameter linearly, to correct for zero point vibrational energy. This procedure yields a cell lattice parameter of 11.72 Å. In the Supplementary Material, the calculated positions of all oxygen and hydrogen atoms are given. It is worth mentioning that, in comparison with the empty clathrate, the guest molecule produces a slight reduction of the lattice parameter, which can be explained by the attraction between the guest molecules and the host lattice. Although repulsive forces play a decisive role in the stabilization of the clathrate structure by preventing its collapse (particulary under pressure), the attractive character of the guest–host interaction at the equilibrium geometry is supported by the reduced density gradient analysis discussed below.

The average distances obtained for the equilibrium zero-pressure structure are: 1.171 Å for the C–O bond, 0.999 Å for the O–H covalent bond, 1.711 Å for the hydrogen bonds and 2.710 Å for the O–O intermolecular distance. In the case of the free CO_2_ molecule, the calculated bond distance is 1.172 Å and the experimental value is 1.162 Å [41]. For the ice I_*h*_ phase, our calculated values for the O–H covalent bond, O–H hydrogen bond, and O–O distance are, respectively, 1.001 Å, 1.701 Å and 2.702 Å. The corresponding experimental values are 1.004 Å, 1.747 Å and 2.751 Å at 60 K [42]. These numbers highlight the accuracy and consistency of our computational methods, and the structural similarity between clathrate hydrates and ice. The carbon atom in the CO_2_ molecule lays slightly off the geometrical center of the cage. This has also been observed experimentally [8], with the experimental offset being around 0.05 Å for the P cages and 0.14 Å in the case of the H cages.

The volume partition yields a volume of 151.54 Å^3^ for the P cage and 212.46 Å^3^ for the H cage. In comparison, the empty hydrate gives 152.39 Å^3^ and 214.44 Å^3^, respectively. We observe again the reduction induced by the guest–host attractive interactions. Both cages decrease in volume when the guest molecule is introduced, but the volume reduction is slightly smaller in the case of the P cage due to its smaller size compared to the H cage, which makes its water framework less compressible.

### 3.2. Equation of State. Microscopic Partition

Pressure effects on the *sI* structure of the CO_2_@H_2_O clathrate are quantitatively described by the Vinet EOS parameters collected in Table 1. The analogous data for the P and the H cages are also provided. The cage EOS parameters are interesting because they may be transferable to other clathrate structures displaying the same cage geometries.

**Figure 3:**
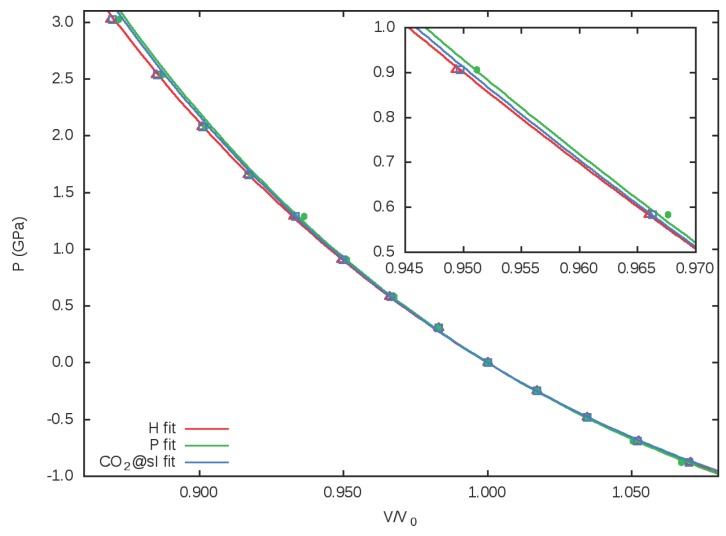
Vinet EOS and data points for the unit cell, P and H cages. The corresponding normalized volumes, $V/V_{0}$, with respect to pressure, *p*, are plotted.

Table 1 shows that the bulk moduli for the clathrate and its two cages are very similar, with the H cage being slightly more compressible than the P cage. This is purely a size effect, since the P cage is the smaller and the least compressible of the two. In Figure 3, the normalized volume versus pressure curves illustrate the similar compressibilities for the bulk and the two cages. Overall, the behavior of the unit cell corresponds to a weighted average of the two types of cages. The fact that the three curves are hardly differentiated means that this clathrate responds homogeneously under hydrostatic pressure regarding cage volumes. Whether this homogenous behavior in the volume is due to an average of the distortions of different cage distances or it corresponds to an isotropic response of the whole clathrate framework will be discussed in the following subsection. However, it is clear from these results that the volume reduction of the P and H cages in the *sI* structure of CO_2_@H_2_O under pressure approximately follows the same behavior as the bulk. Our EOS parameters for the bulk can be compared with recent theoretical results of Jendi et al. [43]. As discussed in this study, the incorporation of attractive dispersion interactions induce a reduction in the lattice parameter (and volume) and, hence, enhance the value of $B_{0}$. This fact explains the slight discrepancies with respect to our results in the calculated values of *a* = 11.90 Å, *V* = 1687 Å^3^, and $B_{0}$ = 10.40 GPa obtained in the PBErev calculations of Jendi et al. [43]. Notice also that their value for $B_{0}^{\prime }$ = 6.27 is slightly higher than ours, thus cancelling the discrepancy in the pressure–volume diagram.

### 3.3. Cage Deformation

Depending on the guest molecule and/or the applied pressure, the local geometry of clathrate cages may show different distortions. To determine the deformation of a cage, we assume that the distances from its geometrical center (G) to the centers of all its equivalent faces (F) are the same. We propose descriptors to measure the deviation of the cage shape from its regular symmetric geometry using these distances. In the case of the P cage, we define the deformation index of the cage ($D_{P}$) as the difference between the largest and shortest G–F distance. $D_{P}$ is zero if the polyhedron is regular, and it becomes larger the greater the distortion of the cage. In the case of the H cage, two deformation indexes are required. The first deformation index ($D_{H}$) is analogous to $D_{P}$ and is defined in the same way except it uses the distances to the twelve pentagonal faces of the H cage. The isotropy index ($I_{H}$) is defined as the ratio between the average of the two G–F (hexagonal) distances and the average of the twelve G–F (pentagonal) distances. Values below 1 indicate oblate shapes of the H cage, whereas if $I_{H}$ is greater than 1, then the cage has a prolate shape. Changes of this index with pressure are related to an anisotropic distortion of the H cage.

In Table 2, we collect the calculated values of these indexes for different pressures in the empty and fully occupied clathrates. $D_{P}$ and $D_{H}$ are averaged values over the two ($D_{P}$) and six ($D_{H}$) cages of each type. Comparing both systems at a given pressure, for example at p∼ 0 GPa, we see that, in the case of the empty clathrate, the low values of the $D_{P}$ and the $D_{H}$ indexes illustrate the regular symmetry of the cages. However, under small pressure, cages are affected by the CO_2_ guest molecules, leading to cage distortions with $D_{P}$ ($D_{H}$) values being more than ten (two times) the corresponding values in the empty clathrates, respectively. These $D_{P}$ and $D_{H}$ values do not provide information on the structural isotropy of the clathrate since they indicate differences in distances and do not involve any specific direction in the crystal. Notice, however, that the isotropy index $I_{H}$ remains almost constant whether the H cages are empty or fully occupied. This result agrees with the homogeneous character of the pressure-induced distortions discussed above.

Although $D_{P}$ and $D_{H}$ indexes increase with pressure due to the fact that the available space is progressively reduced, the planarity of the H cages remains more or less the same with the $I_{H}$ index around 0.82. Thus, H cages show a similar oblate (flattened) shape in the whole pressure range for both empty and saturated clathrates. This means that the relative distortion of the H cages along the *a* and *c* axes is almost negligible. We can conclude that the distortions in the host lattice are induced by either the guest molecules or pressure, but the planarity of the H cages is a characteristic feature of the *sI*-type structure. Our results are in agreement with the Zener anisotropy factor $A_{Z}$ calculated by Jendi et al. [43]. $A_{Z}$ is a ratio involving the three elastic constants of a cubic crystal: $A_{Z}$ = $2c_{44}$/($c_{11}-c_{12}$), and its departure from 1 is a measure of the elastic anisotropy of the system. Shimizu et al. [44] noticed that, although cubic polymorphs of molecular crystals such as CO_2_-I, H_2_O-VII, SH_2_ and CH_4_ display $A_{Z}$ values between 2 and 6, the experimental value of this parameter for the *sI* phase of CH_4_@H_2_O is around 1.2 at room conditions. The calculated values of Jendi et al. [43] are 0.99 and 0.88 for CH_4_@H_2_O and CO_2_@H_2_O, respectively. The elastic isotropy of the cubic hydrate clathrate phase is explained in terms of its peculiar structural features [43,44]. Our results support this distinctive behavior of the *sI* phase. The isotropic behavior of this structure can be related to the non-directional character of the weak electrostatic-type host–guest interactions, discussed below.

### 3.4. Internal Rotations, Conformations

**Figure 4:**
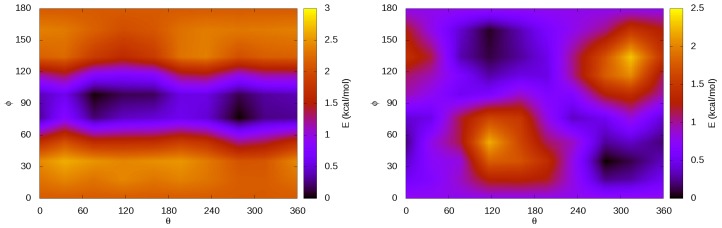
Energy map for the H cage (**left**) and the P cage (**right**). *ϕ* is the angle with respect to the *c*-axis and *θ* is the angle with respect to the *a*-axis. Darker colors correspond to more stable orientations.

**Figure 5:**
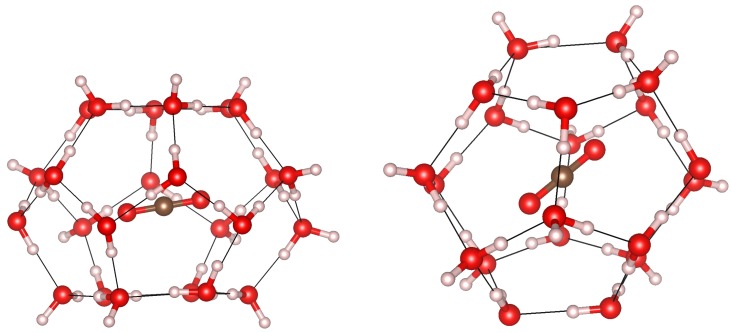
Optimized configurations of the CO_2_ molecule inside the H (**left**) and P (**right**) cages.

Our results for the calculations performed varying the orientation of the CO_2_ molecule inside each of the two cages are illustrated in Figure 4. In the case of the P cage, we find that CO_2_ has its lowest energy orientation when the internuclear axis is pointing to the center of one of the pentagonal faces. In the case of the H cage, the CO_2_ molecule lays with the three atoms parallel to the hexagonal faces ($\phi =90^{{}^{\circ}}$) in its most stable configuration. These geometries are represented in Figure 5.

In addition to the lowest energy configuration, we find that, in the H cage, CO_2_ presents a plane of allowed atomic movements where the molecular axis is parallel to the hexagonal faces. This observation is in agreement with previous diffraction measurements [7,8]. In these experiments, the Rietveld refinement of the diffraction data showed a better fit when the oxygen atoms of the CO_2_ molecule inside the H cage are treated as a torus. These results are in agreement with our rotation plane centered at $\phi =90^{{}^{\circ}}$ with a spread of around 20° in both clockwise and anti-clockwise directions. In reference [8], it was also determined that the most frequent orientation of the molecule, which is the most stable one, is not totally parallel to the hexagonal faces but has a tilt angle between 6.5° and 14.4°. After detailed exploration of Figure 5, we observe that the dark blue color associated with the lowest energy (*ϕ* = 90°) orientation of CO_2_ in the H cage has a narrow width that is in agreement with the experimental tilt angle.

We also estimated the libration frequency related to the hindered rotation orthogonal to the free rotation plane in the H cage. The energetic barrier for this libration is around 3 kcal/mol and the associated libration frequency is *ν* = 45 cm^−1^. As far as we know, this value is reported here for the first time. Such a low value is in agreement with the relatively low energy of the barrier involved. Remarkably, this frequency and its intensity could be used as a probe to detect the formation of CO_2_@H_2_O clathrates and their occupation.

### 3.5. Cage Occupation

**Figure 6:**
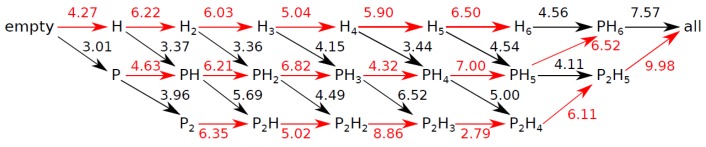
Released energy (in kcal/mol) after the occupation of a P (**black** arrows) or H (**red** arrows) cage of *sI* CO_2_@H_2_O.

In Figure 6, we present an arrow diagram reporting the energy (in kcal/mol) released when one extra CO_2_ molecule is added to a P (black arrow) or H cage (red arrow) of the unit cell of CO_2_@H_2_O.

The figure shows that full occupation is energetically favorable, with a total energy released after saturation of 46.09 kcal/mol. In addition, the stabilization due to the occupation of the cages by CO_2_ increases with pressure by a value around 3 kcal/mol at 1 GPa. Therefore, pressure that appears favors clathrate hydrate saturation. So far, there is only one known empty hydrate structure expected to be stable at negative pressures [45]. Overall, our static calculations allows us to state that, provided there is an excess of CO_2_ molecules in the environment, the process of filling this *sI* phase is energetically favorable, although entropic effects need to be considered as well.

The figure also shows that the difference in energy between the H and P cages is small but significant, and we predict that CO_2_ will occupy the H cages preferentially. This is in agreement with previous experiments [8] at ambient pressure and 173 K, where it was found that all H cages were occupied but P cages were only occupied in a 0.71 ratio. Experimentally, the partial occupation of P cages can be explained by the slower kinetics related to the small size of the cage and the lower energetic preference of CO_2_ for it. Defects in real clathrate systems may also prevent saturation, as well as the entropy difference between the guest molecule in gas phase and inside the clathrate. In the case of the H cage, our calculations show that the energy released is higher than in the case of the P cage, 6.03 kcal/mol vs. 4.56 kcal/mol. This is in contrast with the results we obtained when the guest molecule is methane [46]. Methane does not show a meaningful preference for one type of cage over the other, and releases a similar amount of energy when P or H cages are occupied. However, the total amount of energy released (47.04 kcal/mol) is practically the same as with CO_2_ upon saturation with methane molecules [46]. This greater preference for the H cage in the case of CO_2_ guest molecules can be explained due to the different interactions with the host network arising from its linear configuration. The fact that the symmetry of CH_4_ is higher than CO_2_ produces a more isotropic interaction of the methane with the water molecules in the P and H cages.

We can also calculate the density of the structures with different CO_2_ occupations from the equilibrium cell geometries. The empty clathrate has a density of 0.86 g/cm^3^, and the fully occupied clathrate has 1.24 g/cm^3^, with partially filled structures showing intermediate values: one occupied H or P cage raises the density up to 0.91 g/cm ^3^, and when all H cages are occupied, the density goes up to 1.15 g/cm^3^. In the case of full P-cage occupation, the density is 0.95 g/cm^3^. These observations are in good agreement with previous studies [8,10,47,48]. As mentioned above, the experiments do not find saturation of all cages and the observed total occupation yields values ranging from around 7.1 to 7.42 cages occupied per unit cell (of a total of 8) [8]. The density change related to the cage occupation is one of the contributions to the change in buoyancy of these compounds [8]. The pressure-induced changes could critically affect the internal distribution of materials within planetary objects. Effects of this influence and behaviour under pressure of several ice phases and brines are shown in Reference [49], where an alternative multilayered structure of Ganymede is proposed. In our case, the density of the fully occupied clathrate hydrate is comparable to ice V.

### 3.6. Guest–Host Interactions

**Figure 7:**
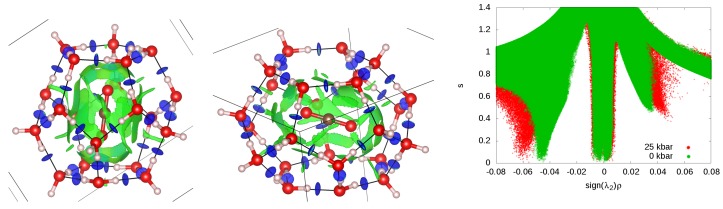
(**left, center**): Isosurface of the RDG (*s* = 0.5). The color is determined by the value of the density times the second eigenvalue (−0.03 is **blue** and 0.03 is **red**). The P cage (**left**) and the H cage (**center**) are shown, both with the most stable orientation of the CO_2_ molecule. (**right**): plot of the RDG vs. sign$(\lambda_{2})\rho$ in the whole crystal at two different pressures.

We examine the type of guest–host interactions and their strength using the NCI technique [33,34,35]. In Figure 7 we display two different NCI representations. The first one contains the isosurface of the reduced density gradient function (RDG = *s* = 0.5) inside the P and H cages of the CO_2_@H_2_O crystal. The second representation shows how *s* changes with sign$(\lambda_{2})\rho$. Different colors of the isosurface follow the coding given in Section 2: red represents repulsive interactions, blue represents attractive interactions and green represents dispersion forces.

As expected, strong hydrogen bonds are found between water molecules, while between host and guest molecules, the green color reveals dispersion interactions. In spite of the linearity of the carbon dioxide molecule, the green areas are equally distributed around it at the equilibrium geometry, confirming the isotropic (non-directional) character of the interaction. Notice that the leading multipole in a CO_2_ molecule is a quadrupole, which interacts electrostatically with the water framework. In Figure 7 (right), we see that the peak at −0.04 associated with the existence of H-bonds is shifted towards a higher absolute value of sign$(\lambda_{2})\rho$ when pressure is increased from 0 to 2.5 GPa. This means that the strength of the H-bonds is enhanced in the clathrate structures when pressure is applied. At the same time, the domains associated to the dispersion interactions become slightly wider. This indicates that those domains are populated by higher electron density values. Since dispersive interactions have been shown to correlate with the volumes of the NCI regions [50], this observation can be interpreted as an increase in the strength of guest–host interactions. No interactions between guest molecules belonging to different cages was found.

### 3.7. Vibrations

We performed calculations of the normal mode vibrational frequencies of a finite gas-phase cluster comprising two adjacent clathrate cages, one P and one H. Due to the large number of atoms, (222 atoms, 660 vibrational modes), a complete vibrational analysis is difficult to carry out. For our purposes, we focus on the symmetric stretching (SS) and antisymmetric stretching (AS) modes of the CO_2_ molecule due to their importance, in Raman and IR studies of the *sI* phase of the CO_2_@H_2_O crystal, respectively. These local modes of the guest molecule are easy to identify in our calculations. We obtain zero pressure values of 1368 cm^−1^ and 1358 cm^−1^ for the SS mode in the P and H cages respectively, being 2433 cm^−1^ and 2422 cm^−1^ the corresponding values for the AS mode. When compared with our calculated frequencies for the free molecule, 1282 cm^−1^ (SS) and 2283 cm^−1^ (AS), we observed that these stretching modes suffer a blueshift upon encapsulation of ∼80 cm^−1^ for the SS frequency and around ∼150 cm^−1^ for the AS one. This blueshift is higher by around 10 cm^−1^ in the case of the P cage compared to the H cage. We can interpret these results in terms of two contributions. The strongest effect is due to the confinement of the guest molecule in the *sI* crystal structure. This effect is subsequently modulated with a cage size correction. The smaller the cage, the higher the frequency of both the SS and AS modes. The cage size correction is small since, in spite of a difference in the volumes of the P and H cages of more than 60 Å^3^ (see Table 1), the blueshift is only around 10 cm^−1^ higher for the P cage than for the H cage.

This interpretation is supported by the calculated pressure effects on both the SS and AS frequencies. Increasing *p* up to 2 GPa produces an overall volume reduction of about 15 Å^3^ and 20 Å^3^ for the P and H cages, respectively (see Figure 3). In the case of the small cage (P), this pressure leads to an additional blueshift of only 5 cm^−1^ for the SS frequency and around 10 cm^−1^ for the AS mode. The corresponding values for the H cage are smaller and the same for both modes (2 cm^−1^). We have found difficulties comparing our calculated values with those obtained experimentally because in our calculations we can model the Fermi resonance that appears when the Raman spectra of CO_2_ is measured, since this is an anharmonic effect. Nevertheless, if we compare with the Raman experimental values of the doublet formed around 1278 and 1384 cm^−1^ for the CO_2_@H_2_O system [51], we find a reasonable agreement with our calculated frequencies of the SS mode in the P (1368 cm^−1^) and H (1358 cm^−1^) cages.

## 4. Conclusions

In this article, we performed density-functional theory calculations using both periodic and cluster approximations in the cubic *sI* phase of the CO_2_@H_2_O clathrate hydrate, and analyzed its structure, behavior under pressure, and chemical interactions. Equation of state (EOS), CO_2_ saturation and guest vibrational frequencies were calculated at different pressures. The decomposition of the EOS in cage contributions and the analysis of cage distortions allow us to conclude that the guest molecule causes a loss in the ideal symmetry of the empty structure, but the distortions are largely isotropic. This is further confirmed when pressure effects on the structure are examined. H cages have an oblate shape, but the isotropic $I_{H}$ index reveals that pressure does not make the H cages more oblate. A study of the orientation of the guest molecule inside the two types of cages finds that the most stable position of CO_2_ lies close to the center of the cages with only a free rotation plane in the H cage, the one parallel to the hexagonal faces. The addition of guest molecules to unoccupied clathrate cages is always energetically favorable. In contrast with methane, the H cages are energetically favored compared to the P cages when CO_2_ is used as the guest molecule. Using the NCI analysis, we have described the intermolecular interactions that bind the clathrate hydrate structure. Dispersion interactions are a key part of the host–guest stabilization. Our calculations of vibrational frequencies show that there is a blueshift upon encapsulation in the stretching modes of the CO_2_ molecule due to a constraining effect from the crystal structure. Cage effects and pressure should be understood as minor factors to the overall increasing of these local frequencies.

## Figures and Tables

**Table 1:** Vinet EOS parameter $V_{0}$, $B_{0}$ and $B_{0}^{\prime }$ for the CO_2_ hydrate, and its P and H cages.

	*V*_0_ (Å^3^)	*B*_0_ (GPa)	$B_{0}^{\prime }$
CO_2_@H_2_O	1577.87	15.42	5.59
P cage	151.54	15.75	5.46
H cage	212.46	15.41	5.09

**Table 2:** Pressure dependence of cage deformation parameters for the empty and full occupied CO_2_@H_2_O clathrate (see text for symbols). $D_{P}$ and $D_{H}$ in Å.

Empty				Full			
*p* (GPa)	*D_p_*	*D_H_*	*I_H_*	*p* (GPa)	*D_p_*	*D_H_*	*I_H_*
3.60	0.0106	0.0663	0.8193	3.37	0.4035	0.3081	0.8184
3.01	0.0100	0.0679	0.8194	3.04	0.3688	0.2174	0.8188
2.53	0.0098	0.0675	0.8193	2.56	0.3457	0.1945	0.8188
2.07	0.0094	0.0671	0.8193	2.10	0.3366	0.1863	0.8188
1.73	0.0092	0.0634	0.8189	1.68	0.3172	0.1788	0.8188
1.33	0.0096	0.0633	0.8188	1.31	0.2561	0.1590	0.8191
0.97	0.0107	0.0633	0.8188	0.93	0.2543	0.1531	0.8190
0.65	0.0118	0.0635	0.8188	0.61	0.2297	0.1471	0.8190
0.36	0.0131	0.0639	0.8188	0.33	0.2061	0.1367	0.8189
0.10	0.0141	0.0640	0.8187	0.02	0.1936	0.1314	0.8189
−0.14	0.0152	0.0642	0.8187	−0.22	0.1815	0.1256	0.8189
−0.35	0.0164	0.0645	0.8187	−0.46	0.1708	0.1216	0.8189
−0.52	0.0167	0.0627	0.8185	−0.67	0.1626	0.1155	0.8188
−0.68	0.0150	0.0604	0.8183	−0.86	0.1536	0.1112	0.8187

## Supplementary Materials

The following are available online at www.mdpi.com/1996-1944/9/9/777/s1: a cif file containing the unit cell parameters and atomic positions of all atoms of the full CO_2_@H_2_O hydrate is provided.
